# 2158. An outbreak of carbapenem-resistant *Acinetobacter baumannii* at a tertiary hospital in Ethiopia

**DOI:** 10.1093/ofid/ofad500.1781

**Published:** 2023-11-27

**Authors:** Mulatu G Adbaru, Esayas K Gudina, Arne Kroidl, Andreas Wieser

**Affiliations:** Jimma University and Ludwig Maximilian University, Jimma, Oromiya, Ethiopia; Jimma University, Jimma, Oromiya, Ethiopia; Division of Infectious Disease and Tropical Medicine, University Hospital (LMU), Munich, MUNICH, Bayern, Germany; Head of Parasitology, Max von Pettenkofer-Institut for Hygiene and Medical Microbiology, LMU Munich, Munich, Bayern, Germany

## Abstract

**Background:**

Since there are no effective alternative treatments, health professionals in resource-constrained countries struggle to treat infections caused by microorganisms those are resistant to carbapenem antibiotics. The purpose of this study was to determine the magnitude of carbapenem-resistant *Acinetobacter baumannii* isolated from patients with surgical site infection.

**Methods:**

A cross-sectional study was conducted among patients who underwent surgical procedures at Jimma Medical Center. Two wound swab samples were collected from patients who had clinical evidence of surgical site infection (SSI) and processed at JMC microbiology laboratory. All *A. baumannii* species were confirmed by MALDI TOF MS and the carbapenem-resistant isolates were further characterized using PCR.

**Results:**

Of 1205 participants enrolled in the study, more than one-fourth (312) of them showed clinical evidence of SSI. A couple of wound swabs were collected from 83.3% (260) of the patients who had SSI for microbiological analysis. Of those, 72%, (189/260) were culture positive with 252 isolates and 36 different bacterial species. *Acinetobacter baumannii*, (20.2%; 51) were the second most frequent bacteria and showed the highest resistance to carbapenem antibiotics. The PCR analysis revealed *bla*OXA-51 60.8% (31/51) was the predominant carbapenemase-encoding gene followed by *bla*NDM 23.5% (12/51), and *bla*VIM, *bla*OXA-23, and *bla*OXA-58 (15.7%; 8 each) genes. About 37.3% (19) of the strains were co-harboring two or more resistance-encoding genes for carbapenem antibiotics.

**Conclusion:**

It is concluded that *A. baumannii* is one of the predominant bacteria causing SSIs. The resistance genes *bla*OXA-51 and *bla*NDM-1 were highly prevalent in the strains. In light of this finding, it is recommended that current antimicrobial resistance surveillance programs and infection prevention practice and control measures should be re-evaluated.

**Disclosures:**

**All Authors**: No reported disclosures

